# A Citizen Science Approach: A Detailed Ecological Assessment of Subtropical Reefs at Point Lookout, Australia

**DOI:** 10.1371/journal.pone.0163407

**Published:** 2016-10-05

**Authors:** Chris Roelfsema, Ruth Thurstan, Maria Beger, Christine Dudgeon, Jennifer Loder, Eva Kovacs, Michele Gallo, Jason Flower, K-le Gomez Cabrera, Juan Ortiz, Alexandra Lea, Diana Kleine

**Affiliations:** 1 University of Queensland Underwater Club (UniDive), University of Queensland, St. Lucia, Queensland, Australia; 2 Remote Sensing Research Centre, School of Geography Planning and Environmental Management, University of Queensland, St. Lucia, Queensland, Australia; 3 School of Life and Environmental Sciences and Centre for Integrative Ecology, Deakin University, Warrnambool, Victoria, Australia; 4 School of Biological Sciences, University of Queensland, St. Lucia, Queensland, Australia; 5 School of Biomedical Sciences, University of Queensland, St. Lucia, Queensland, Australia; 6 Reef Check Australia, Reef Check Foundation Ltd (Australia), Brisbane, Queensland, Australia; 7 ARC Centre of Excellence for Coral Reef Studies and School of Biological Sciences, University of Queensland, St. Lucia, Queensland, Australia; 8 CoralWatch, Queensland Brain Institute, University of Queensland, St. Lucia, Queensland, Australia; University of Bologna, ITALY

## Abstract

Subtropical reefs provide an important habitat for flora and fauna, and proper monitoring is required for conservation. Monitoring these exposed and submerged reefs is challenging and available resources are limited. Citizen science is increasing in momentum, as an applied research tool and in the variety of monitoring approaches adopted. This paper aims to demonstrate an ecological assessment and mapping approach that incorporates both top-down (volunteer marine scientists) and bottom-up (divers/community) engagement aspects of citizen science, applied at a subtropical reef at Point Lookout, Southeast Queensland, Australia. Marine scientists trained fifty citizen scientists in survey techniques that included mapping of habitat features, recording of substrate, fish and invertebrate composition, and quantifying impacts (e.g., occurrence of substrate damage, presence of litter). In 2014 these volunteers conducted four seasonal surveys along semi-permanent transects, at five sites, across three reefs. The project presented is a model on how citizen science can be conducted in a marine environment through collaboration of volunteer researchers, non-researchers and local marine authorities. Significant differences in coral and algal cover were observed among the three sites, while fluctuations in algal cover were also observed seasonally. Differences in fish assemblages were apparent among sites and seasons, with subtropical fish groups observed more commonly in colder seasons. The least physical damage occurred in the most exposed sites (Flat Rock) within the highly protected marine park zones. The broad range of data collected through this top-down/bottom-up approach to citizen science exemplifies the projects’ value and application for identifying ecosystem trends or patterns. The results of the project support natural resource and marine park management, providing a valuable contribution to existing scientific knowledge and the conservation of local reefs.

## Introduction

Marine environments, particularly near-shore reefs, are under severe threat from human impacts [1–3]. However, data collection is generally resource intensive in terms of cost and expertise required. Thus, despite the importance of reefs, their typical stress responses, recovery trajectories, and fine-scale spatial differences are unknown in most places. The result is that large areas of even relatively accessible shallow water reef habitats remain unmonitored, leaving data gaps that could be filled by citizen science programs [4, 5].

Volunteer participation has become an increasingly significant component of ecological research projects, particularly where high levels of observation effort are typically required to assess biodiversity, or to understand temporal and spatial community dynamics [6–12]. Citizen science projects (defined as the involvement of volunteers in research) [12] have proliferated in the terrestrial realm over the last decade, and more recently marine-focused projects are increasing in popularity [10]. For example, globally active organisations such as Reef Check Australia (RCA) and CoralWatch facilitate the community to participate in visual censuses of benthic and fish communities [4, 6, 9].

The involvement, skills and knowledge required of volunteers in marine and coastal citizen science projects varies, ranging from projects that use minimal training of volunteers, to training-intensive projects. For example, CoralWatch provides a simple approach that does not require specific SCUBA training as it can be done walking, by snorkel and/or on SCUBA to undertake surveys [9, 13]. RCA, on the other hand, provides an approach that requires three to four days of comprehensive training in invertebrate, substrate, fish and reef impact identification and survey protocols [14]. Various research and management projects have engaged volunteers to create habitat maps of underwater environments, which are often also an important component of RCA and CoralWatch surveys [15, 16].

The organisation of citizen science projects typically involves either a top-down or bottom-up approach [17]. A top-down approach is characterised by a strong science and/or management-driven organisation where scientists, non-government or government managers organise and train volunteers to collate large amounts of information to support specific research and management questions. Examples of these include RCA, CoralWatch, or the Great Barrier Reef Marine Park Authority’s Eye on the Reef [6]. A bottom-up approach is characterised by citizen science projects that are driven by community groups, who have a particular goal or information they wish to gather and assess, and who subsequently seek scientific advice to run the project, e.g. Fiji Local Marine Managed Areas [18].

While concerns have been raised about the quality and compatibility of data collected by volunteers [19–21], citizen science programs have succeeded in initiating data collection on a scale that would not be possible using trained scientists alone [12]. Many programs also document their quality assurance and quality control procedures, providing a guideline for appropriate data application. In addition, citizen science programs build community capacity, facilitate community understanding on environmental issues, and increase environmental stewardship [9, 10, 22]. As a result they have significantly increased capacity to monitor and observe changes in accessible near-shore marine environments [14].

Subtropical reefs occur around the world in bio-geographical transition zones from tropical to temperate regions [1]. Historically, little research has been conducted in subtropical regions compared to tropical reef habitats, and even today monitoring efforts remain limited [23, 24]. Well-developed subtropical reefs, coral/algal communities on rocky platforms, fringe the east coast of Australia at the southern end of the Great Barrier Reef, below the tropic of Capricorn [23, 25, 26]. Subtropical reef systems are important to study due to unique and highly transitional species assemblages that are an excellent indicator of climate change impacts [27]. In addition to the overarching threat of climate change, subtropical reefs are considered at risk of environmental change from exploitation, and habitat destruction including coastal development and pollution [24]. Divers and fishers, who frequent these sites, can be a source of information on environmental change [28–30], helping to augment data collected from science and management agencies.

Reefs around Point Lookout, North Stradbroke Island, South East Queensland, support a high variety of fish, invertebrates, corals and algae. The area has exceptional biodiversity value for megafauna, hosting an aggregation site for zebra sharks (*Stegostoma fasciatum*) [31], manta rays (*Manta alfredi*) [32] and the critically endangered grey nurse shark *(Carcharias taurus)*[33]. Additionally, the area is a ‘hot spot’ for the seasonal migrations of humpback whales (*Megaptera novaeangliae*) [34]. Due to both inherent ecological value and ecosystem services supported, this area is critical to monitor and appropriately manage.

Earlier citizen science projects at Point Lookout reefs produced a baseline ecological assessment in 2001, and since 2009, annual RCA surveys have been conducted at some of the Point Lookout dive sites [35–37]. Although these previous assessments provide a significant amount of information, informed natural resource management decisions are difficult to make as knowledge gaps remain. Firstly, the 2001 baseline study can be considered outdated, as a number of factors may have influenced the flora and fauna since then. These include natural impacts (e.g. 2011 and 2013 Brisbane Floods), increased recreation pressure due to an increased population, and changes in marine park zonation in 2009. Additionally, the annual RCA surveys are limited to four sites, and are therefore unable to capture potential seasonal changes or indicate changes at others sites. Neither the McMahon study, nor the RCA surveys have generated detailed georeferenced maps of all the Point Lookout dive sites that can support management and monitoring of the area. This is compounded by the current limited resources available to marine park agencies to fund staff to conduct surveys. Hence citizen science is perfectly poised to fill these gaps. Through volunteer citizen science schemes, data information gaps such as detailed georeferenced habitat maps for reefs, comparison of habitat and biodiversity among sites with differing topography and exposure, and seasonal changes in benthos, fish, invertebrates and reef impacts can be addressed [38].

The aim of this study was to demonstrate the broad range of marine ecological and spatial data that can be produced using an approach that incorporates both top-down (researchers) and bottom-up (divers/community) engagement aspects of citizen science and provide an ecological assessment at a small spatial scale (100-1000s m). This study provides an example of how marine citizen science can encourage collaboration, support a diversity of increased scientific knowledge and offer an improved knowledge platform for management decisions. To demonstrate the applicability of such a citizen science project approach, we focused on the analysis and discussion of three critical project elements. Firstly, we outline the process by which the project was implemented and the methods used to ensure accuracy of the data collected. Secondly, we provided an assessment of the spatial distribution of benthic habitat characteristics including benthic organism composition, habitat maps and human impacts. Thirdly, using examples of fish community and substrate composition we identified seasonal change and spatial differences in marine assemblages.

## Methods

### Project Initiation, Leadership and Citizen Science Training

The Point Lookout citizen science project was initiated by the University of Queensland Underwater Club (UniDive). UniDive has ±350 members, predominantly students and academic researchers both with and without marine science backgrounds, varying in age, gender and dive experience. UniDive has a history of undertaking citizen science-based projects [35, 37], dive training and specialised SCUBA diving courses and/or marine survey techniques (e.g. Reef Check Australia (RCA) and CoralWatch).

Volunteers were trained using standardised data collection protocols (based on RCA and CoralWatch protocols), which were imparted by volunteer marine scientists and/or dive instructors, also members of UniDive. The training program included presentations on: coral reef ecology; survey protocols; buoyancy control; identification and biology of coral, algae, substrate, fish, and invertebrates; mapping and, data analysis. Practical training was conducted in-water and included: identification of indicator species and reef impacts, buoyancy control, underwater mapping and survey technique.

### Study Sites and Survey Overview

The Queensland Parks and Wildlife Service (QPWS), The Department of National Parks, Sport and Racing, Queensland Government, Brisbane, Queensland, Australia, granted permission for our assessment of the near shore Point Lookout Reefs included in this study.

**Fig 1:**
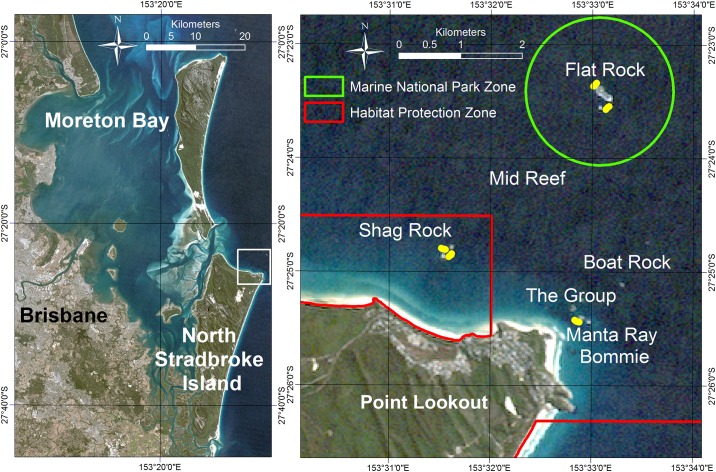
Location of the study site within Moreton Bay Region, South East Coast Queensland, Australia (Left panel). Point Lookout dive sites offshore of North Stradbroke Island are indicated by the white box, (Right Panel) the offshore dive sites at Point Lookout, North Stradbroke Island with Marine Park zonation marked in red and green. The study transect locations are indicated in yellow. Source background image: Landsat Thematic Mapper 5, USGS (http://earthexplorer.usgs.gov).

Surveys and mapping were conducted for the Point Lookout dive sites at Flat Rock, Shag Rock and Manta Ray Bommie, all adjacent to the northeastern tip of North Stradbroke Island (Fig 1). The study sites are characterized by submerged rocks overgrown with algae and/or coral. The rocky reefs are influenced by southeast trade winds and swells that round the headland, as well as the East Australian current which brings in warmer waters and marine larvae from the tropics [27]. Mid Reef and Boat Rock were not included due to water depth and currents.

The ecological assessment was conducted along semi-permanent transects that each consisted of 3 x 20 m segments each 5 m apart at approximately 10 m below chart datum (Fig 1). Semi-permanent transects were marked for the duration of the project with a concrete block, that was removed at the conclusion of the study. Semi-permanent transects will be referred to in this paper as transect(s). Flat Rock and Shag Rock each had two transect sites which were planned to closely replicate the same areas surveyed in 2001 [37]. A single transect site was placed at Manta Ray Bommie. Surveys were carried out four times over a 12-month period in order to capture potential seasonal changes in the marine flora and fauna. Differences in assemblages at different times of year were conducted, with sampling times corresponding to each of the main seasons—spring, summer, autumn and winter. It would require more sampling to establish the full validity of these differences to represent mean seasonal variability, which was beyond the scope of this study. All seasonal surveys were completed in a single weekend where possible (Table 1).

**Table 1:** Seasonal marine flora and fauna surveys conducted in 2014 for each transect location at Point Lookout, Australia.

Site	Latitude	Longitude	Summer	Autumn	Winter	Spring
**Flat Rock West**	27° 23’ 21” S	153° 33’ 02”	22nd Feb.	3rd May	2nd Aug.	25th Oct.
**Flat Rock East**	27° 24’ 33” S	153° 33’ 09”	22nd Mar.	3rd May	2nd Aug.	25th Oct.
**Shag Rock West**	27° 25’ 48” S	153° 32’ 32”	22nd Feb.	3rd May	19th July	25th Oct.
**Shag Rock East**	27° 25’ 52” S	153° 32’ 36”	22nd Feb.	3rd May	19th July	25th Oct.
**Manta Ray Bommie**	27° 25’ 26” S	153° 33’ 51”	22nd Mar.	4th May	19th July	30th Oct.

The methods used for surveying and mapping followed similar protocols in-line with previous sampling methodologies, to enable comparative studies in the future [35, 37]. In short, the ecological assessment incorporated an adapted RCA survey method [4] and CoralWatch Health chart surveys [9]. Mapping methods were based on georeferenced photo transects accomplished by a towed GPS floating at the surface [35, 39].

### Ecological Assessment Methods and Quality Control

The ecological assessment recorded benthic cover, the abundance of indicator fish and invertebrates, and reef impacts along each of the transects, based on RCA methodology [4].

Volunteer competence was assessed by a theoretical identification exam and an in-water survey exam (85% pass rate required for theory and practical). To further improve data quality during collection and analysis, volunteers reviewed indicator species before each survey weekend, while underwater data sheets included pictures to aid identification. Volunteer marine scientists also reviewed the data after each survey and after each quarterly survey season to check for errors or inconsistencies. Feedback and training for volunteers continued throughout the project to build and maintain identification skills.

The benthos was assessed using two methods, firstly, with point intercept counts where the benthic category was recorded at each 0.5 m segment along the transect, and secondly, using a georeferenced photo-transect method [39]. Indicator fish family and fish species, and target invertebrates (Table 2), were recorded over a 5 m wide belt along each transect. Indicator taxa were chosen as indicators of reef health (Table 2), such as those commonly caught by recreational or commercial fisheries (e.g. cod), targeted by aquarium collectors (e.g. banded coral shrimp, clown fish), or, those that provide an indicator of pollution (e.g. specific algae species).

**Table 2:** Indicator categories for fish, invertebrates, substrate and reef impacts, modified from comparable surveys [4, 35, 36].

Fish Families (*spp*)	Fish Species (*spp*)	Invertebrates	Substrate	Impacts
Angel *(Pomacanthidae)* Butterfly *(Chaetodontidae)* Cardinal *(Apogonidae)* Cods/Groupers *(Serranidae)* Damsel *(Pomacentridae)* Emperors *(Lethrinidae)* Goat *(Mullidae)* Leatherjackets *(Monocanthidae)* Lion/Stone *(Scorpaenidae)* Morays *(Muraenidae)* Parrot *(Scarridae)* Pipefish/Seahorse *(Sygnathidae)* Porcupine *(Diodontidae)* Puffer *(Tetraodontidae)* Rabbit *(Siganidae)* Snappers *(Lutjanidae)* Surgeon *(Acanthuridae)* Sweetlips *(Haemulidae)* Stingrays (*Dasyatididae*) Trigger *(Balistidae)* Wrasse *(Labridae)* Wobbegong (*Orectolobidae*)	Moorish Idol *(Zanclus cornutus)* Keyhole Angelfish *(Centropyge tibicen)* Barred Soapfish *(Grammistes fasciatus)* Flagtail Triggerfish *(Rhinecanthus aceculatus)* Black-saddled Toby *(Canthigaster compressa)* Bluespot Butterflyfish *(Chaetodon plebeius)* Guenthers (Crochet) Butterflyfish *(Chaetodon guentheri)* Orange (Klein’s) Butterflyfish *(Chaetodon kleinii)* Bigscaled Scalyfin *(Parma oligolepis)* Indopacific Sergeant *(Abudefduf vaigiensis)* Buffalofish *(Parma polylepis)* Coral Sea Gregory *(Stegastes gascoynei)* Blue damsels *(Pomacentrus coelestis*, *P*. *pavo)* Black bar devil *(Plectorglyphidodon dickii)* Sunset + Moon wrasse *(Thalassoma lutescens*, *T*. *lunare)* Cleaner Wrasse *(Labroides dimidiatus)* Red Morwong *(Cheilodactylus fuscus)* Magpie Morwong *(Cheilodactylus vestitus)* Happy Moments Rabbitfish *(Siganus fuscescens)* Sixplate Sawtail *(Prionurus microlepidotus)* Silver drummer *(Kyphosus sydneyanus)*	Anemones (*various spp*.) Trochus (*Trochus niloticus*) Triton (*Charonia tritonis*) Collector Urchin (*Tripneustes* spp.) Long Spine Urchin (*Diadema* spp.) Pencil Urchin (*Phyllacanthus parvispinus*) *Drupella* spp. Giant Clam (Tridacna spp.) Crown of Thorns (*Acanthaster plancii*) Banded Coral Shrimp (*Stenopus hispidus*) Sea Cucumbers: Prickly Green Fish (*Stichopus chloronotus*) Prickly Red Fish (*Thelenota ananas*) Pink fish (*Holothuria edulis*)	**Hard Coral:** *Plate*, *Encrusting*, *Foliose*, *Branching*, *Massive* **Soft coral:** *Zoanthids*, *Leathery*, *Ornate* **Substrate:** *Rock*, *Rubble*, *Sand*, *Silt*, Recently killed coral with turf algae or crustose coralline algae **Algae:***Ulva* spp., *Dictyota* spp., *Halimeda* spp., *Lobophora* spp., *Laurencia spp*., *Caulerpa* spp., *Asperogopsis* spp., *Chlorodesmus* spp., *Turbinaria* spp., *Sargasum* spp.,*Lyngbya* spp.	**Coral Stress:** *Bleaching*, *Disease* **Damaged Coral:** *Anchor*, *Unknown* **Scars:** *Unknown*, *Crown of thorn*, *Drupella* **Macro-debris:** *Fish Nets*, *Fish Lines*, *Trash*

Fish species were picked to represent a range of functional groups (e.g. herbivory, corallivory), and tropical or subtropical zoogeographic affiliation (determined using FishBase; [40]), with consideration for ease of identification by the volunteer divers (Table 2).

Impact surveys recorded the number of occurrences of specific physical impacts (e.g. broken coral), coral bleaching, coral disease, the occurrence of rubbish or ‘other’ impacts over a 5 m wide belt along the transect, adhering to RCA’s Impact Survey Protocol [4].

The ecological assessment was conducted during a staggered 40–50 min dive per buddy team. The first team to enter the water deployed the transect tape and acquired photos of notable features. After a ten minute interval the fish team entered the water to ensure fish numbers had recovered to that prior to transect tape deployment, a time frame determined previously [41]. After this at successive 5 min intervals, invertebrate then reef impact teams entered and the last team in the water retrieved the transect tape at the completion of the survey.

Approximately 100 volunteers participated in the academic component of the training of which 50 participated in the dive surveys. Experience level of the divers varied, but 90% had more than 100 dives and were certified as Rescue Diver or higher. No volunteer diver was beginner level Open Water and no diver had less than 50 dives. 75% of the volunteer divers participated in more than one survey trip whilst 40% of the volunteer divers participated in all surveys.

Coral health was assessed using coral health charts developed by CoralWatch [9, 13]. Individual coral colonies were selected randomly along each transect (to a maximum of 20 colonies). For each colony the diver used the coral health chart to record the darkest and lightest colour present, through a colour scale ranging from 1(white)-6(dark), thus giving an approximate assessment of coral health, where a light colour is considered unhealthy (e.g. bleached).

### Habitat Mapping

Mapping of the three sites was conducted to depict the main morphological features (e.g. major substrate types, gullies and ridges) and provide a reference for future ecological surveys, planning and zoning of the sites (e.g. installation of mooring buoys). Feature mapping was undertaken by a roving survey of each site, to a maximum depth of 20 m, and recorded characteristic features using methods based on previous work [35, 39]. Feature location was mapped by cross-referencing the time each feature was recorded or photographed, with GPS data recorded by a floating GPS towed by the observer. Based on previous work by the authors the offset between the diver and the surface was within 2–7 m [35]. Rough sketches and notes about the features and water depth changes were made underwater to help characterise the environment.

Imagery from Google Earth were imported into the mapping program Q-GIS (www.qgis.org) and used as a backdrop to manually digitize the outline of the rock-sand interface that was clearly visible. All georeferenced photographs that documented each of the reefs were plotted on top of the imagery at the respective locations to provide additional information in addition to the sketches and notes from the survey. GPS tracks and the georeferenced photographs overlaid on the satellite imagery were then used to manually digitize and label distinctive point or line features (e.g. swim through, wall, gully). Water depth contours were manually digitised based on the diver’s observations, expert knowledge, echo soundings from previous surveys, and expert interpretation of the satellite imagery. As open source software Q-GIS, CPCe and GPS-Photo Linking software (e.g. DNR GPS), and the Google Earth and Landsat Imagery are freely accessible they provide ideal tools for citizen science-based projects.

### Data Analysis

For all categories and indicators, abundance was calculated. For the belt surveys, this was per area covered, and for the point intercept surveys this was calculated as a percentage of the transect. To evaluate if there are differences in substrate structure, reef impacts and marine fauna between sites and seasons we used linear models including sites, seasons and their interrelationship. All analyses were done using the R statistical program [42]. For significant models we performed Tukey’s post-hoc multiple comparison tests for the response variable per site, per season. Where the assumptions for normality and homoscedasticity were not fulfilled, we applied a log+1 transformation. Fish, families were grouped into five trophic groups for the assessment: predators, herbivores, invertivores, corallivores and omnivores. Multivariate PERMANOVA analyses were conducted using Primer-E software [43].

Reef impacts were normalized for the coral cover present at the site being assessed (percentage of substrate cover). Normalising reef impact abundance with coral cover acknowledges that many reef impacts specifically affect corals. As such, the ratio of coral cover to impact abundance should be considered when interpreting reef impact data.

## Results

### Habitat Mapping

**Fig 2:**
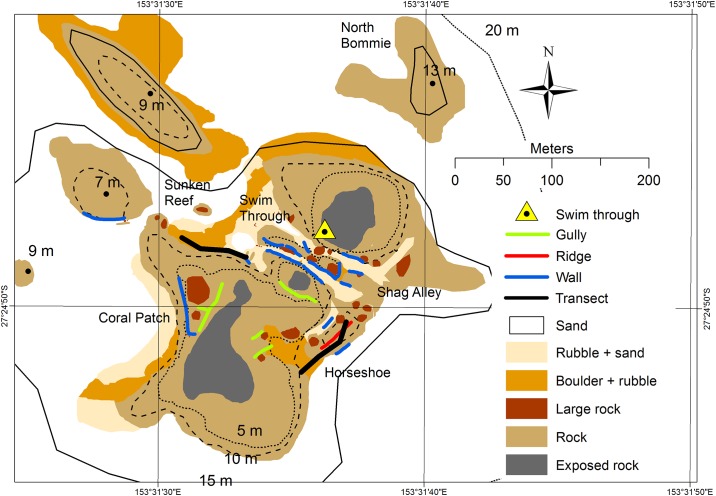
Example habitat map. The habitat map created for Shag Rock, Point Lookout, North Stradbroke Island, Australia using GIS data and significant feature recording of the benthos. GIS data are available online [44].

Habitat maps were created for Shag Rock, Manta Ray Bommie and Flat Rock. These provide information on the characteristics of the different sites in regards to a simplified substrate composition and water depth (Fig 2) [44].

Flat Rock was the furthest from shore and covered the largest area (~65 Ha), followed by Shag Rock (~12 Ha) and Manta Ray Bommie (~4 Ha). The deepest water was found near Flat rock (35 m), followed by Shag Rock (18 m) and Manta Ray Bommie (16 m).

### Data Analysis

#### Substrate

Differences between sites and seasons for the benthic cover were assessed using a PERMANOVA analysis with Bray-Curtis distances and 999 permutations. The assumption of homogeneity of dispersion (permdisp) was met for both factors and significant effects were found for both sites (Pseudo-F = 5.2927, p = 0.002) and seasons (Pseudo-F = 5.0072, p = 0.002). Post-hoc pairwise tests showed that the variation in sites was significant between Flat Rock East and Shag Rock East (t = 2.314, p = 0.04) primarily due to a higher proportion of turf substrate at Shag Rock East (SIMPER analysis). Additionally, variation was significant between Flat Rock East and Manta Ray Bommie (t = 3.9754, p = 0.041) primarily driven by a higher proportion of hard coral at Flat Rock East and non-living substrate at Manta Ray Bommie. Marginal significance was found between Flat Rock West and Shag Rock West (t = 2.908, p = 0.05) with the former site having a higher proportion of algae. The major variation in seasons was between spring with both summer (t = 2.3291, p = 0.038) and winter (t = 2.1205, p = 0.027) primarily driven by a higher proportion of turf in summer and winter.

**Fig 3:**
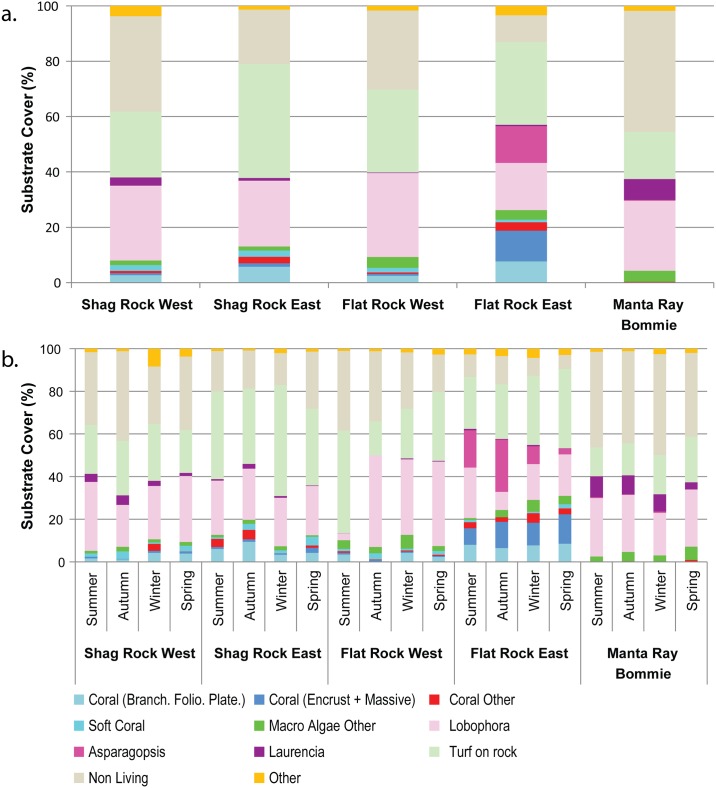
Benthic composition of the Point Lookout transect sites derived from a photo transect approach. (a.) Substrate cover type for each of the five transects at, Shag Rock, Flat Rock and Manta Ray Bommie averaged over the four seasons, and (b.) Substrate cover type per site for each season.

The five transects (Fig 1) varied in percentage and type of coral cover (Fig 3a), where the values were derived using the photo transect approach as it was considered more consistent when compared to a point intercept approach. The highest coral cover occurred at Flat Rock East, averaging 22.8% across all seasons, and mainly consisting of encrusting (11.1%) and branching type hard corals (7.7%), with a low level of soft coral cover (0.9%). Shag Rock East had the second highest coral cover (14.4%), dominated by branching or foliose type corals (5.7%), with some encrusting (2.3%) and soft coral (2.4%) cover. The lowest average coral cover (<1%) was observed at Manta Ray Bommie.

Macro algae (e.g., *Lobophora*, *Asparagopsis* and *Laurencia*) and turf algae accounted for the highest percentage cover (>50%) of the substrate types at all sites (Fig 3a). Turf algae and *Lobophora* were observed in similar amounts across all sites, while *Asparagopsis* and *Laurencia* were most abundant at Flat Rock East (13.3%) and Manta Ray Bommie (1.2%) respectively. Interestingly, fluctuations in algal cover were observed seasonally (Fig 3b). *Asparagopsis* and *Laurencia* were most prevalent from summer through to autumn, with the lowest levels of these algae observed in spring (Fig 3b). Decreased levels of *Lobophora* were observed at both Flat Rock sites in summer or autumn (west and east sites respectively), while turf algae coverage remained constant at all sites, over the year (Fig 3b).

#### Indicator Invertebrates

**Fig 4:**
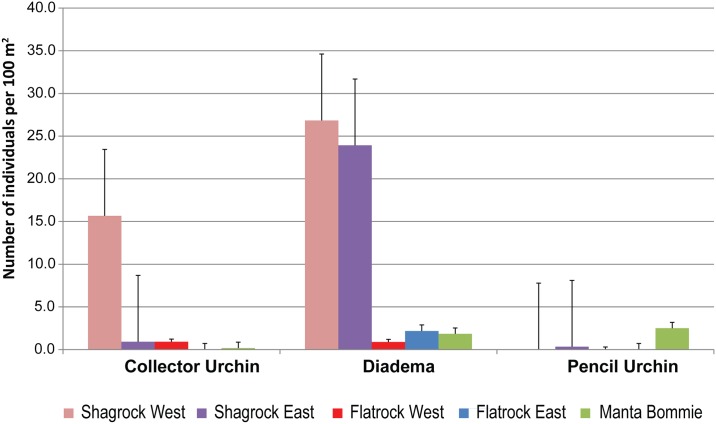
Indicator invertebrates—urchins. Average abundance of indicator urchins for all Point Lookout sites. Error bars indicate standard deviation.

Three species of urchins were recorded as indicator invertebrates and were the most common of all of the invertebrates at each of the sites and therefore data analyses were conducted on these invertebrates only. All three species showed strong statistically significant differences for Sites, and an interaction effect between Sites and Season. However, single factor analysis on Season showed no significant differences suggesting that this pattern was driven primarily by site. *Diadema* (*Echinothrix diadema* and *Diadema* spp.) urchins were the most common and were observed with significantly greater frequency at the Shag Rock sites than at the other sites (Fig 4; Table 3). Collector urchins had a significantly higher abundance at Shag Rock West than the other sites. Conversely pencil urchins were significantly more abundant at Manta Ray Bommie.

**Table 3:** Statistical tables for representative invertebrate linear models testing Site, Season, and their interaction (Site:Season).

Species	Transformation	Factor	df	p-value
*Diadema*	Log+1	Season	3	0.002
		Site	4	**2.2E-16**
		Season:Site	12	**2.04E-05**
Collector urchin	Log+1	Season	3	0.002
		Site	4	**7.91E-16**
		Season:Site	12	**4.21E-07**
Pencil urchin	None	Season	3	0.001
		Site	4	**8.45E-10**
		Season:Site	12	**2.74E-06**

Banded coral shrimp (*Stenopus hispidus*), giant clams (*Tridacna* spp.) and lobsters (*Panulirus* spp.) were rare, averaging abundances of <1 per 100m^2^ at all sites, whilst indicator sea cucumbers, *Trochus niloticus* and *Triton* shells were not observed at any sites (data not shown).

#### Reef Impacts

**Fig 5:**
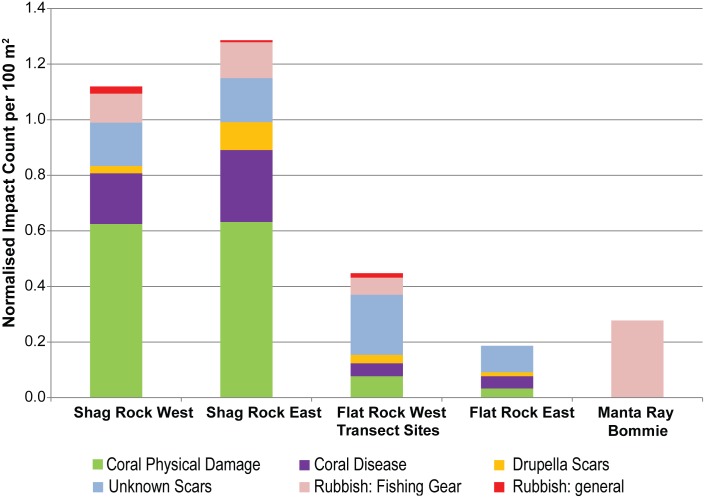
Reef Impacts for each of the Point Lookout dive sites. Normalised cumulative abundance of reef impacts weighted by coral cover per site across surveys.

Reef impacts on corals at both Shag Rock sites were significantly higher than that observed at the Flat Rock sites (Table 4). The two Shag Rock sites had high instances of observed physical damage to coral and coral disease relative to all other sites examined (Fig 5). Coral scars (from *Drupella* snails and unknown causes), coral disease, physical damage and rubbish were recorded consistently at both Shag Rock, and both Flat Rock locations (Fig 5). Unknown scars were predominant at Flat Rock West whilst Manta Ray Bommie displayed the greatest amount of fishing-associated rubbish (Fig 5). Negligible reef impacts on substrate and no coral damage was recorded at Manta Ray Bommie (Fig 5).

**Table 4:** Post-hoc multiple comparison of reef impacts per site. Sites were statistically different (Pseudo F = 5.2309, p = 0.018).

	Shag Rock West	Shag Rock East	Flat Rock West	Flat Rock East	Manta Ray Bommie
Shag Rock West					
Shag Rock East	0.704				
Flat Rock West	0.040**	0.069*			
Flat Rock East	0.051*	0.060**	0.060**		
Manta Ray Bommie	0.251	0.329	0.594	0.243	

** represents statistical significance (p< 0.01),

* represents marginal significance (p< 0.05).

**Fig 6:**
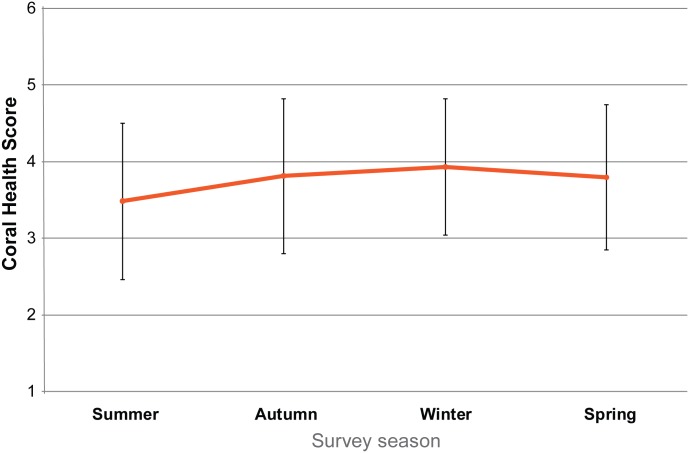
Coral Health at Point Lookout. The average coral health observed for each season based on CoralWatch coral health chart readings at the five transect sites at Point Lookout, Australia. The error bars represent the range of the average dark to average light scores that were recorded.

For the period of observation, the recorded coral health was relatively stable with no obvious bleaching (Fig 6), with a score of 6 considered healthy. Overall, the lightest scores were observed in summer, when the water temperature was higher. As data were consistent and similar for the five transects, they were amalgamated to create an average for the Point Lookout dive site region. Fig 6 shows that the average score ranges from 3.5 in summer to 4 in winter.

#### Reef Fish Communities

**Fig 7:**
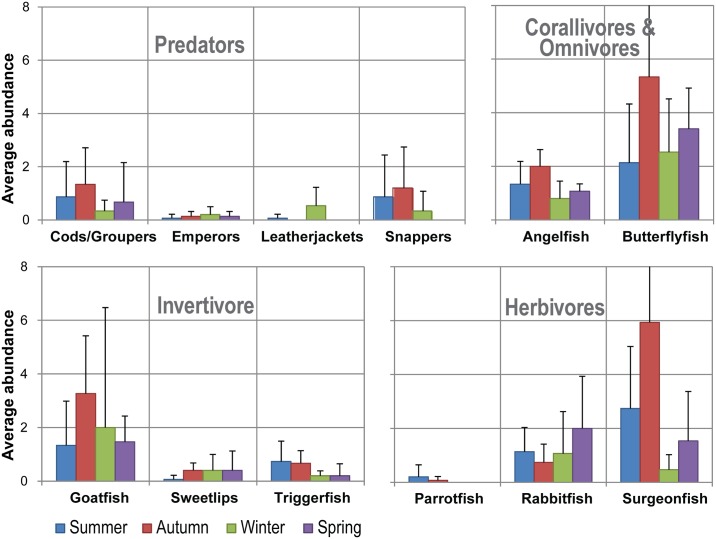
Seasonal changes of different trophic groups of fish. Seasonal abundance (+- SD) of predators, corallivores and omnivores, invertivores and herbivores averaged for each season at the Point Lookout Dive Sites.

All five sites exhibited a diversity of fish families and species (Fig 7), with damsel and wrasse families in the highest abundance—annual combined site averages of 85.9 x 10^2^ for damsels and 9.9 x 10^2^ for wrasses (data not shown).

Variation was observed seasonally with subtropical fish groups observed more commonly in colder seasons, whilst tropical groups such as parrotfish were comparatively rare year-round (Fig 7).

Seasonal changes were apparent for the different trophic groups of fish. Herbivores and invertivores were observed primarily in summer and autumn (respectively—surgeonfish and parrotfish, Fig 7: lower right panel; and, goatfish and triggerfish, Fig 7: lower left panel). Predatory families were not abundant overall, although there were slight observed increases in autumn (Fig 7: upper left panel). Similarly, tropical and subtropical corallivores and omnivores were most abundant in autumn (Fig 7: upper right panel).

Changes of fish assemblages were apparent both among sites and across seasons. For example, butterflyfish assemblages lacked seasonality but differed among sites (0.0040, Table 5), with significantly different communities between Flat Rock West and the two Shag Rock sites (S1 Table). A significant seasonal difference was observed for damselfish (p = 1.566E-06, Table 5), which was most apparent in autumn (S1 Table). This was consistent across all sites. A similar seasonal relationship was discovered for surgeonfish (p = 0.0002, Table 5), with a significant difference between autumn and the other seasons (S1 Table). Rabbitfish displayed no significant difference across either sites or seasons (Table 5).

**Table 5:** Statistical tables for seasonal fish analyses Linear Models testing Site, Season, and their interaction (Site:Season).

Family	Transformation	Factor	df	p-value
Butterflyfish	Log+1	Season	3	0.1549
		Site	4	**0.0040**
		Season:Site	12	0.1264
Damselfish	None	Season	3	**1.566E-06**
		Site	4	**0.0211**
		Season:Site	12	0.0563
Surgeonfish	Log+1	Season	3	**2.179E-05**
		Site	4	0.07725
		Season:Site	12	0.07249
Rabbitfish	None	Season	3	0.9414
		Site	4	0.1398
		Season:Site	12	0.5179

## Discussion

In contrast to other citizen science programs in South East Queensland, this project was unique in its approach. Instead of using a top-down approach to recruit volunteers, this project employed a bottom-up approach initiated by a community group of SCUBA divers, with high levels of support from professional scientists.

This study aimed to demonstrate the range of data that can be produced by citizen scientists and to illustrate that citizen science surveys can capture seasonal shifts and site-specific differences in flora and fauna in a marine environment. The project provided sufficient numbers of volunteers to collect data at a scale and level of detail not performed before for the habitats in this region, while also maintaining consistency with and complementing existing programs. Surveys were performed across multiple sites and seasons, for a diversity of species groups using a robust training program that incorporated a variety of different survey techniques and required volunteers to demonstrate their skills to a high standard. Survey training and quality control measures were provided by dive club members with a background and/or experience in marine science. The success of the volunteer project has been demonstrated through the ability to discover and describe morphological and biological variability across space and time for a dynamic coastal subtropical reef system. The results provide examples of how citizen engagement can be successfully applied to surveys of subtropical reef ecosystems, and the value of citizen-collated data to science, both as baseline survey data and in contributing to existing survey activities (e.g., RCA).

Although the project was based upon a 2001 ecological baseline assessment of the same area, methods have improved. Additionally, the 2001 data is potentially out-dated due to natural or anthropogenic impacts. The major improvements incorporated into the current project included more detailed training of the survey volunteers, RCA protocols were followed to record an increased number of invertebrate and reef impact indicators, coral health data was documented based upon CoralWatch protocols, and the capability for underwater digital photography enabled georeferenced photo surveys for benthic assessment and habitat mapping.

### Detecting Spatial and Temporal Variation

The substrate data showed spatial differences between habitats on the different subtropical reefs. Each of the transect sites are subject to discrete exposure regimes and are located different distances from shore, leading to varying conditions at small spatial scales (hundreds to thousands of metres). Such variability is almost never quantified by scientific studies, which tend to examine assemblage processes at spatial scales of tens of kilometres or more [25].

Shag Rock, Flat Rock and Manta Ray Bommie exhibited very different benthic characteristics, both within and among sites. For example, Flat Rock East exhibited high levels of coral cover compared to other sites, especially encrusting coral, reflecting its greater exposure to waves and wind. In contrast, Manta Ray Bommie exhibited the lowest level of coral cover and the highest abundance of non-living material, such as rock and sand. Although the abundance of the dominant algal types (turf algae and *Lobophora*) changed little over time, seasonal variations in algae composition were observed for less prevalent algal types (*Asparagopsis* and *Laurencia*).

Invertebrate abundance differed between sites, which may reflect the differences in dominant substrate cover and the subsequent availability of suitable habitat. Flat Rock sites had a low abundance of all sampled invertebrates compared to Shag Rock sites. The less exposed Shag Rock sites were dominated by three-dimensional habitat consisting of branching, foliose and plate corals, which are known to provide more crevices and therefore, presumably, more suitable habitat for invertebrates [45, 46]. Some invertebrate indicators were not recorded during the surveys. These included *Trochus*, *Triton* and sea cucumbers, likely because these species are most commonly associated with tropical habitats.

Fish families commonly exhibited seasonal differences, with the exception of predatory families. Tropical and subtropical corallivore and omnivore families were more abundant in autumn, while invertivore families exhibited their lowest abundance in spring, and herbivorous families were observed primarily in summer and autumn. Interestingly however, no differences were detected among sites or seasons in the herbivorous rabbitfish, despite observed seasonal fluctuations in algal substrates. It is possible that the preferred algae targeted by rabbitfish did not change seasonally. It is also possible that volunteers did not record rabbitfish reliably, as this group often roams rapidly across transects. The low numbers of predatory fish recorded may arise due to the length of transects being inadequate for sampling species with larger territories. Larger predatory fish tend to be rare and are often diver phobic as their potential predators are larger animals. They may also display patchy distributions on reefs. Dedicated surveys for larger fish species such as timed swims rather than transect lines may be more effective for predatory or large fish species [47].

Physical coral damage can be due to natural causes such as storms, or anthropogenic factors such as boat anchoring, divers, snorkelers and fishing [48]. The greatest amount of physical damage occurred at the Shag Rock sites. Of this, the most commonly recorded category of damage was ‘other’ for which the cause is unspecified, but could include boat anchors, divers or storms. Compared to other sites, Shag Rock is closer to the shore, it has no moorings, and it is a legalised fishing zone. These factors may encourage more frequent visits by divers and fishers compared to the other sites. Additionally, the dominant coral growth form was branching coral. Compared to, for example, encrusting coral that is dominant at Flat Rock, branching corals are more vulnerable to physical impact. In combination, the branching coral at the Shag Rock sites is therefore more exposed to the potential for damage, and more susceptible to damage.

Discarded fishing lines were recorded at Flat Rock during these surveys. Flat Rock has been designated as a marine national park zone precluding anchoring, and the area was closed to fishing under the Fisheries Act for Grey Nurse Shark 2012. Therefore, this suggests that the fisheries closure is not fully enforced, although it is possible (if unlikely) that the discarded fishing materials date from pre-2012. Manta Ray Bommie, a site that is open to fishing, exhibited zero physical damage to coral, reflecting the largely sandy and rocky substrate and low coral cover. However, volunteer divers recorded larger quantities of discarded fishing material at Manta Ray Bommie than at the other two sites.

The environmental conditions (e.g. wind, swell, tides, and temperature) varied during the surveys due to normal weather patterns, which would influence both the flora and fauna present and the ability of divers to observe particular species. Certainly, both visibility and currents varied and will have contributed to intra-season differences. Hence, ongoing seasonal surveys are valuable to distinguish between real seasonal change and observer error, and would provide us with a greater knowledge of what species are present or absent at different times of the year.

### Limitations of Volunteer Surveys

Whilst a high number of volunteers were imperative for successful data collection at the scale proposed, this does present potential issues regarding the reliability of collated data (also discussed elsewhere, e.g. [19, 21]). For example, some variation was present in the results that could not be explained through seasonality alone. One example is that changes in hard coral cover were observed between seasons that would not have been expected given the slow growth rate of such species. While the potential for bias was minimized through the use of robust training procedures, it is likely this variation arises from data being collected by different divers across different surveys.

Observer error in volunteer data is probably most important to consider for fish because of their transient behaviour, giving volunteers less time to identify and count them correctly. Repeated seasonal surveys, and perhaps comparisons with expert data, will be required to confirm our fish results. Surveys where photos can be taken such as for substrate and reef impacts, offer additional opportunities for data validation as it is then possible to incorporate post survey data checking processes for quality control.

Due to the large number of volunteers involved in the project and the challenges with making notes underwater, clarity of notes was not always consistent resulting in errors in post-survey data entry. Hence, it’s important to have: easy to use underwater datasheets; experienced and/or trained divers that can control their buoyancy; and data entry and validation soon after completion of the dives.

## Conclusions

This study demonstrated how citizen scientists can synthesize a broad range of marine ecological and spatial data by using an approach that incorporates both bottom-up (divers/community) and top-down (researchers) engagement aspects of citizen science. Volunteers were involved in all aspects of the project: applying for funding to support their citizen science project, conducting surveys, entering and quality-controlling data and publication. The results of this project add to the growing citizen science literature by demonstrating the type, quality and quantity of data that can be delivered when volunteers are provided with the tools, training and support to actively participate in ecological studies.

Fine-scale ecological patterns on subtropical reefs were quantified at the target sites for the first time, enhancing the knowledge about these sites for managers, decision makers, and recreational users. The ecological assessment and analysis revealed that even reef sites in close proximity (hundreds to thousands of metres) could contain different microenvironments demonstrating a degree of habitat variation over small spatial scales. The differences in environments are potentially due to proximity to land, prevailing currents, swell and winds, in addition to other topological factors. The study further revealed that these microenvironments vary in composition over time, which may be explained by seasonal changes in environmental conditions on subtropical reefs [1, 49].

Regular monitoring of local reefs would improve understanding of ecological changes and enable scientists and management agencies to better understand if these are due to natural variations or caused by external factors such as fishing, pollution or physical damage. The monitoring would provide data to support management decisions that would otherwise not be collected due to a lack of resources within local marine authorities, hence confirming the importance of citizen science.

Citizen science is not just about utilising volunteers to collate scientific data. It is also about engaging the community to create awareness of the importance of the natural environment, to instil a sense of ownership and pride, and to promote co-learning opportunities about the need to maintain and conserve our ecosystems in the face of increasing pressures. The success of this citizen science project was highlighted through: the inclusion of the results and findings [44, 50] in resource management and marine park planning by the local authorities; the eagerness of the participants to be involved in future projects; and peer review of this manuscript prepared by the volunteers.

Additional detail of the project can be found online in regards to: methods [51], report results [50], raw data [52] and digital data for maps [44].

## Supporting Information

S1 Table (PDF)
