# Supplementary material for: A Citizen Science Approach: A Detailed Ecological Assessment of Subtropical Reefs at Point Lookout, Australia
Source: PLoS One. 2016 Oct 5;11(10):e0163407. doi: 10.1371/journal.pone.0163407 (PMC5051685; doi:10.1371/journal.pone.0163407)
Supplement: S1 Table — (PDF) [file pone.0163407.s001.pdf]

S1 Table. Analysis of variance of significant models.

Family	Factor	Coeff	p-value
Butterflyfishes	Flat_Rock_West-Flat_Rock_Eas	0.4492982	0.5884541
(log+1)	Manta_Ray_Bommie-Flat_Rock_East	0.1015718	0.9973177
	Shag_Rock_East-Flat_Rock_	0.4964636	0.4913607
	Shag_Rock_West-Flat_Rock_East	0.6194558	0.2696896
	Manta_Ray_Bommie-Flat_Rock_West	0.5508700	0.3854406
	**Shag_Rock_East-Flat_Rock_West**	**0.9457618**	**0.0253080**
	**Shag_Rock_West-Flat_Rock**	**1.0687540**	**0.0082515**
	Shag_Rock_East-Manta_Ray_Bommie	0.3948918	0.6992631
	Shag_Rock_West-Manta_Ray_Bommie	0.5178840	0.4485312
	Shag_Rock_West-Shag_Rock_East	0.1229922	0.9943774
Damselfishes	**Spring-Autumn**	**-99.400000**	**0.0000396**
	**Summer-Autumn**	**-64.933333**	**0.0104633**
	**Winter-Autumn**	**-96.066667**	**0.0000713**
	Summer-Spring	34.466667	0.3224935
	Winter-Spring	3.333333	0.9983393
	Winter-Summer	-31.133333	0.4124560
Surgeonfishes	**Spring-Autumn**	**-1.0493601**	**0.0022700**
(log+1)	**Summer-Autumn**	**-0.6915394**	**0.0748936**
	**Winter-Autumn**	**-1.3447479**	**0.0000673**
	Summer-Spring	0.3578207	0.5787179
	Winter-Spring	-0.2953878	0.7165026
	Winter-Summer	-0.6532085	0.1015445
